# New York City HIV superbug: fear or fear not?

**DOI:** 10.1186/1742-4690-2-14

**Published:** 2005-03-02

**Authors:** Stephen M Smith

**Affiliations:** 1 Section of Infectious Diseases, Department of Medicine, Saint Michael's Medical Center, New Jersey, USA; 2 Department of Preventive Medicine and Community Health, The New Jersey Medical School, Newark New Jersey 07102, USA

## Abstract

On February 11, 2005, the New York City Department of Health and Mental Hygiene announced that a city resident had recently been infected with a multi-drug resistant form of HIV and rapidly progressed to AIDS. The Health Commissioner, Thomas R. Frieden, called for increased vigilance against this new strain. Is this situation an emerging crisis or simply an unusual case report of rapid HIV progression?

On February 11, 2005, New York City (NYC) health officials announced the discovery of a "rare strain of multi-drug resistant HIV that rapidly progresses to AIDS." According to the NYC Department of Health and Mental Hygiene, a man in his mid-40s was diagnosed with HIV infection in December 2004. Shortly after his diagnosis, testing, at the Aaron Diamond AIDS Research Center in Manhattan, revealed that his virus was resistant to almost all anti-HIV therapeutics. Further, despite being infected for only 2–20 months, the man had developed AIDS. NYC Health Commissioner Thomas R. Frieden, MD, MPH, stated, "This case is a wake-up call. First, it's a wake-up call to men who have sex with men, particularly those who may use crystal methamphetamine...now, we've identified this strain of HIV that is difficult or impossible to treat and which appears to progress rapidly to AIDS." Dr. Frieden called on this community to help stop the spread of this and other drug resistant strains of HIV. He also called on NYC doctors and the public health system to improve HIV prevention counseling, to perform HIV drug resistance testing among treatment naïve, HIV^+ ^persons, and to improve anti-HIV drug adherence.

At the 12^th ^Conference on Retroviruses and Opportunistic Infections (CROI) in Boston, Drs. David Ho and Martin Markowitz of the Aaron Diamond AIDS Research Center in Manhattan presented clinical and laboratory data regarding the NYC resident[1]. He had tested negatively for HIV-1 antibodies several times before and in May 2003. His total lymphocyte counts during these time points were repeatedly normal. Investigators believe that the NYC resident may have been infected in October 2004, when he, while on crystal methamphetamine, engaged in unprotected, receptive and insertive anal sex with multiple partners. In early November 2004, the NYC resident developed a febrile illness, and then in December 2004, he tested positive for antibodies against HIV. His personal physician, concerned over the possibility of recent acute HIV-1 infection, referred the NYC resident to Dr. Martin Markowitz. At the time of diagnosis, his CD4^+ ^T-cell count was 80 cells/mm^3 ^and it has since fallen to less than 50. The NYC resident meets one criterion for the diagnosis of AIDS; his CD4^+ ^T-cell count is less than 200 cells/mm^3^. His viral load has varied from ~100 K to 650 K/ml. The NYC resident's virus was tested and found to be resistant to all but two anti-HIV drugs, efavirenz (Sustiva^®^) and enfuvirtide (Fuzeon^®^; T20). This high degree of drug resistance existed before the NYC resident was treated with any anti-HIV compound.

Is this case a harbinger of a new epidemic with this superbug or is it just an isolated, forme fruste of HIV infection? No one knows the answer to this question yet, but we do have plenty of data to suggest that the latter is the case. In people naïve to drug therapy, bone fide antiviral resistance is uncommon. A recent USA based study of treatment-naïve patients found that the prevalence of mutations associated with drug resistance was 8.8%[2]. This means 8.8% of the subjects' viruses tested positive by genotyping for 1 or more mutations associated with drug resistance. Having a single mutation associated with resistance does not necessarily make a virus drug resistant. For many drugs, HIV must contain several mutations to become resistant. This fact is true for most protease inhibitors (PIs) and for several nucleoside analogue reverse transcriptase inhibitors (NRTIs). Therefore, the overall level of drug resistance is well less than 8.8% reported in this study. Indeed, in this study, no significant resistance to protease inhibitors was seen. A similar study found the overall prevalence of drug resistance mutations was 8.3%, as also determined by genotyping[3]. However, when viruses containing these mutations were analyzed by phenotyping, only 39% demonstrated decreased reduced drug susceptibility. In other words, less than 3.5% of all isolates had phenotypic resistance.

A commonly held view on why the level of drug resistance is low is that most mutations associated with drug resistance, also decrease viral fitness. Except the K103N reverse transcriptase mutation, which confers resistance to the three approved non-nucleoside inhibitors (NNRTIs), other mutations, associated with high level drug resistance, are thought to significantly decrease viral fitness. Theoretically, in the absence of drug pressure in a newly infected individual, wild-type virus is either selected for during transmission or the transmitted, resistant virus mutates back towards the fitter wild type. The current observation is that the vast majority of viruses in treatment-naïve patients are sensitive to almost all drugs.

In addition to CD4, the virus, isolated from the NYC resident, uses CXCR4 or CCR5 to enter cells[1]. Such viruses, termed dual-tropic, are rarely seen in newly infected individuals. Typically, an R5 virus, which utilize CCR5, is the transmitted type. After years of infection, in approximately 50% of individuals, the viruses' tropism changes from CCR5 to CXCR4[4]. This phenotypic change is associated with an accelerated disease course. People, who are homozygous for the CCR5, 32-bp deletion, do not express functional CCR5 and have a high relative resistance to infection with HIV. In those Δ32 homozygotes, who have become infected with HIV (8 individuals reported), the disease course appears to be more rapid[5]. Most of these individuals had CD4^+ ^T-cell counts less than 300 cells/mm^3 ^at the time of diagnosis. It is unclear why these individuals became infected, while the vast majority of Δ32 homozygotes remain uninfected. Possibly, these 8 individuals have some other aberration, which allows them to become infected with an X4 virus and, in turn, leads to an accelerated disease course. Perhaps, the NYC resident has a similar abnormality, which has lead to an increased rate of CD4^+ ^T-cell depletion.

Also at CROI this year, Drs. Stephen Gange and Alvarez Muňoz from Johns Hopkins University Bloomberg School of Public Health presented models of rapid HIV progression probability, based on two, large prospective cohorts[6]. These studies, the MACS and theWIHS, have been on-going for the past 21 and 11 years respectively and have collected longitudinal data on 391 seroconverters. In the pre-HAART era, the median time to AIDS was 8.3 years. Using the cohorts' data, Drs. Gange and Muňoz estimated the probability of clinical AIDS developing within 6–24 months or a low CD4^+ ^T-cell count existing at the first visit after diagnosis (within the first 9 months of infection). Their model predicts that 7 in 10,000 patients develop clinical AIDS within 6 months of infection. This number increases to 45 in 10,000 after 12 months. Similarly, 10 in 10,000 HIV infected individuals have a CD4^+ ^T-cell count less than 200 cells/mm^3 ^after 4.5–9 months of infection.

Several reports of rapidly progressing HIV infection have been published. The rapid disease course of the NYC resident is rare, but hardly unique. To date, no cluster of rapid progressors has been described. All rapid progressors have been unrelated, either genetically or virologically. While multi-drug resistant (MDR) viruses may be overall less fit compared with wild-type, drug sensitive strains, MDR HIV still causes steady CD4^+ ^T-cell depletion. Therefore, it is highly probable that the NYC resident has a genetic predisposition, which led to rapid progression, rather than a new strain of HIV-1, which is simultaneously super-aggressive and multi-drug resistant.

Our experience at a large, inner city HIV clinic is in agreement with the above data. We do not see rapidly progressing, newly diagnosed individuals. We also do not see MDR HIV in our treatment-naïve patients. Review of our data does not reveal any evidence of MDR virus in persons, who have never been on therapy.

To determine whether this "superbug" has spread to others, the NYC Department of Health is appropriately and aggressively investigating the sexual contacts of the NYC resident. The reason for the NYC Department of Health press release at this early point in the investigation is unclear. In the absence of a documented cluster of patients, should the entire health system react? No, we should wait for more information. I do agree that genotypic resistance testing for treatment-naïve HIV^+ ^patients is prudent, especially when the person is thought to have been infected within the past year. Of course, most patients do not know when they were infected, so we are testing each new patient.

Given the availability of free, rapid testing for HIV in New Jersey, we are strongly encouraging any one with current or previous high-risk behavior to get tested and determine his/her HIV status. The best way to fight this disease is with knowledge: knowledge on one's infection status, knowledge on how to avoid becoming infected, and knowledge on how not to infect some one else. HIV is not the common cold. It is transmitted through well-described behaviors, predominantly sex, especially receptive anal intercourse, and intravenous drug use with shared needles. These behaviors can be modified to reduce or eliminate the risk of contracting HIV. Two recent studies conclude that universal testing for HIV is a cost effective way to combat this infection in the USA [7-9]. Outreach prevention education and widespread testing are probably more effective public health strategies than sensational press releases. Dr. Frieden's call for increased vigilance against drug resistant HIV implies that regular, old-fashioned HIV infection is not horrific enough. Any one who has seen this disease up close knows that is not the case. While we have partially effective therapies and we better understand its pathogenesis, HIV infection in this country, not as life threatening as it once was, remains quite life altering.
